# Cellulose-dependent expression and antibacterial characteristics of surfactin from *Bacillus subtilis* HH2 isolated from the giant panda

**DOI:** 10.1371/journal.pone.0191991

**Published:** 2018-01-31

**Authors:** Ziyao Zhou, Furui Liu, Xinyue Zhang, Xiaoxiao Zhou, Zhijun Zhong, Huaiyi Su, Jin Li, Haozhou Li, Fan Feng, Jingchao Lan, Zhihe Zhang, Hualin Fu, Yanchun Hu, Suizhong Cao, Weigang Chen, Jiabo Deng, Jianqiu Yu, Wenping Zhang, Guangneng Peng

**Affiliations:** 1 The Key Laboratory of Animal Disease and Human Health of Sichuan Province, College of Veterinary Medicine, Sichuan Agricultural University, Chengdu, China; 2 Chengdu Center for Animal Disease Prevention and Control, Chengdu, China; 3 The Key Laboratory of Conservation Biology on Endangered Wildlife of Sichuan Province, Chengdu Research Base of Giant Panda Breeding, Chengdu, China; 4 Institute of Wild Animals, Chengdu Zoo, Chengdu, China; Bharathidasan University, INDIA

## Abstract

Surfactin secreted by *Bacillus subtilis* can confer strong, diverse antipathogenic effects, thereby benefitting the host. Carbon source is an important factor for surfactin production. However, the mechanism that bacteria utilize cellulose, the most abundant substance in the intestines of herbivores, to produce surfactin remains unclear. Here, we used *B*. *subtilis* HH2, isolated from the feces of a giant panda, as a model to determine changes in surfactin expression in the presence of different concentrations of cellulose by quantitative polymerase chain reaction and high-performance liquid chromatography. We further investigated the antimicrobial effects of surfactin against three common intestinal pathogens (*Escherichia coli*, *Staphylococcus aureus*, and *Salmonella enterica*) and its resistance to high temperature (60–121°C), pH (1–12), trypsin (100–300 μg/mL, pH 8), and pepsin (100–300 μg/mL, pH 2). The results showed that the surfactin expressed lowest in bacteria cultured in the presence of 1% glucose medium as the carbon source, whereas increased in an appropriate cellulose concentration (0.67% glucose and 0.33% cellulose). The surfactin could inhibit *E*. *coli* and *Staphylococcus aureus*, but did not affect efficiently for *Salmonella enterica*. The antibacterial ability of surfactin did not differ according to temperature (60–100°C), pH (2–11), trypsin (100–300 μg/mL), and pepsin (100–300 μg/mL; *P* > 0.05), but decreased significantly at extreme environments (121°C, pH 1 or 12; *P* < 0.05) compared with that in the control group (37°C, pH = 7, without any protease). In conclusion, our findings indicated that *B*. *subtilis* HH2 could increase surfactin expression in an appropriate cellulose environment and thus provide benefits to improve the intestinal health of herbivores.

## Introduction

*Bacillus subtilis* is a widely used animal intestinal probiotic that can adapt well living in animal intestines and secrete a variety of carbohydrate hydrolase enzymes and antibiotics to facilitate host diet utilization and pathogen inhibition, thereby balancing the gut microbiome [1, 2]. One of the most powerful antimicrobial peptides secreted by *B*. *subtilis* is the biosurfactant surfactin, which confers strong antipathogenic effects and has diverse biological activities [3, 4].

Previous studies have illustrated that the bacterial carbon source is an important factor affecting the production of surfactin [5]. Although *B*. *subtilis* can utilize a variety of nutrients, including glucose, sucrose, and galactose, for surfactin production [5], the role of cellulose, which is the most abundant substance in the intestines of many herbivores, such as giant pandas, cows, and sheep, remains unknown. Moreover, the species of the host of origin can also influence the selection of animal probiotics [6]. However, to the best of our knowledge, there are no commercial probiotic strain originating from pandas. In our previous study, we found a bacterial model *B*. *subtilis* strain HH2 isolated from the feces of a healthy giant panda; this strain showed a good adaptation to the herbivore intestinal cellulose environment and exhibited several probiotic functions based on transcriptional regulation [7]. However, the secretion and antibacterial effects of surfactin from this probiotic candidate in the presence of high-fiber conditions remain unclear. Therefore, in this study we try to assess the cellulose-dependent expression and antibacterial characteristics of surfactin from *B*. *subtilis* HH2.

## Materials and methods

### Strain and culture medium

Permission to conduct the feces sample collection was granted by the director of China Conservation and Research Center for the Giant Panda and the research ethics committee of Sichuan Agricultural University. *B*. *subtilis* HH2 from fresh feces collected from healthy pandas was isolated and identified in our previous study [8]. Phylogenetic analysis of *B*. *subtilis* HH2 was based on partial 16S rRNA gene and gyrA gene sequences for further identification at the subspecies level. The 16S rRNA gene were amplified by polymerase chain reaction (PCR) using 8F (5′-AGAGTTTGATCATGGCTCAG-3′) and 1492R (5′-ACGGTTACCTTGTTACGACTT-3′) primers with the following cycling conditions: 95°C for 5 min; followed by 35 cycles of 95°C for 40 s, 55°C for 45 s, and 72°C for 2 min; and a final extension for 10 min at 72°C [8]. The gyrA fragments were amplified by PCR using p-gyrA-f (5’-CAGTCAGGAAATGCGTACGTCCTT-3’) and p-gyrA-r (5’-CAAGGTAATGCTCCAGGCATTGCT-3’). Cycling conditions were as follows: 95°C for 5 min; followed by 30 cycles of 95°C for 30 s, 40°C for 1 min, and 72°C for 1 min; and a final extension for 10 min at 72°C [9]. PCR products were sequenced and used to retrieve homologous sequences with the BLAST algorithm in GenBank of NCBI. Six strains of common intestinal pathogens, including *Escherichia coli* (HHEC1, HHEC2), *Staphylococcus aureus* (HHSA1, HHSA2), and *Salmonella enterica* (HHSE1, HHSE2), were also isolated and identified from the feces of healthy adult giant pandas by 16S rRNA.

Glucose medium was slightly modified from previous studies [7, 10] and contained 70 mmol K_2_HPO_4_, 30 mmol KH_2_PO_4_, 25 mmol (NH_4_)_2_SO_4_, 0.5 mmol MgSO_4_, 10 μmol MnSO_4_, 22 mg ferric ammonium citrate, 8 g potassium glutamate, 6 g potassium succinate, 1% glucose, 0.5 mmol CaCl_2_, 5 μmol MnCl_2_, and 1000 mL ddH_2_O at pH 7.2. The three other media used in this study included mixed-1, mixed-2, and cellulose media which were formulated in the same manner as the glucose medium, except that the main carbon sources were 0.33% sodium carboxymethylcellulose plus 0.67% glucose (mixed-1), 0.67% sodium carboxymethylcellulose plus 0.33% glucose (mixed-2), or 1% sodium carboxymethylcellulose (cellulose) instead of 1% glucose.

### Identification of surfactin from the HH2 isolate

The DNA and RNA of *B*. *subtilis* HH2 were extracted using bacterial total DNA/RNA kit (Tiangen Biochemical Technology Co., Ltd, Beijing, China) according to the manufacturer’s instruction. Reverse transcription was performed immediately after RNA extraction using RT Reagent Kit with gDNA Eraser (TaKaRa, Dalian, China).

The surfactin gene *sfp* was detected by polymerase chain reaction (PCR) and reverse transcription (RT)-PCR with the primers F (5′-ATGAAGATTTACGGAATTTATATG-3′) and R (5′-TTATAAAAGCTCTTCGTACGAG-3′) [8, 11]. Cycling conditions were as follows: 95°C for 5 min; followed by 35 cycles of 95°C for 40 s, 55°C for 45 s, and 72°C for 2 min; with a final extension for 10 min at 72°C. PCR products were confirmed by electrophoresis on 1% agarose gel, photographed using Bio-Rad GelDoc XR System (Bio-Rad Laboratories, CA, USA), purified using TIANgel Midi Purification Kit (Tiangen Biochemical Technology Co., Ltd.), and sequenced (Invitrogen Biotechnology Co., Ltd., Shanghai, China). Sequencing results were applied to retrieve homologous sequences using the BLAST algorithm in GenBank of NCBI.

Ten microliters of the solution containing the HH2 strain was added to 1 mL LB medium at 37°C for 16 h for activation from refrigerator and then grown in 100 mL LB medium at 37°C in a shaker at 180 rpm for 18 h, at which time the optical density at 600 nm (OD_600_) was equal to approximately 1. After cultivation, the cells were removed by centrifugation at 4°C, 8,000 rpm for 10 min and the resulting supernatant was sterilized again using bacterial filtration (Φ = 0.22 μm). After adjustedpH to 2.0, the cell-free supernatant was centrifuged again at 4°C, 8000 rpm 20 min. The precipitate was then extracted twice with 10 mL methanol, and then adjusted pH to 7.0 with NaOH. Samples were next centrifuged at 10,000 rpm for 5 min, and the collected organic fraction was further processed using a rotary evaporator at 40°C and diluted to 1 mg/mL. The crude extract of surfactin was analyzed by high-performance liquid chromatography (HPLC; GE Pharmacia AKTA Purifiers 10) using a C-18 column (250 mm × 4.6 mm) at 25°C. Separation was carried out using a H_2_O/trifluoroacetate/acetonitrile (ACN) 0.1% (v/v) solvent system monitored at 210 nm. After an initial 2-min wash with 60% ACN, elution was achieved in 60 min at a flow rate of 1 mL/min from 0% to 100% ACN, followedwash 5 min with 100% ACN [12]. A standard sample of commercial surfactin (Wako Pure Chemical Industries, Ltd., Japan) was treated as described for the crude extract as a control. The crude extract eluent having the same peaks as the standard sample was considered the pure extract. The HPLC sample having the same three peaks was collected by the timeline and used for subsequent analysis.

### Expressional level of surfactin

One milliliter activated *B*. *subtilis* HH2 from LB medium was cultured in 100 mL of the four different media for 18–27 h until reaching an OD_600_ of approximately 1, respectively. The bacteria and surfactin extracts were then collected for analysis of surfactin expression by RT-qPCR and HPLC.

The expression of surfactin mRNA was first measured by RT-qPCR using the following primers: srf-F, 5′-AAAACAGAGTACAGCGACCTT-3′, and srf-R, 5′-AAGCGATAAGCCTTTGCCTTC-3′. For 16S rRNA, which was used as a reference gene, the primers were as the same as those described for bacterial identification. The reaction was performed in a 25-μL reaction mixture containing 5 μL cDNA, 12.5 μL IQ SYBR GREEN Supermix (Bio-Rad Laboratories) and specific primers, with the following conditions: 3 min hot start at 95°C followed by 40 cycles of 95°C for 30 s, 60°C for 30 s, and 72°C for 40 s. Each sample was run in triplicate. Surfactin expression was quantified using the comparative CT method, and data were analyzed by applying the Relative Expression Software Tool (version REST-384) [13].

Surfactin extracted from cells grown in the four media was diluted to 1 mg/mL and then analyzed by HPLC as described above. The areas of the three peaks having the same location as the commercial surfactin solution were calculated to evaluate the peptide moieties.

### Measurement of the antimicrobial ability and pressure resistance of surfactin

We selected three common gut pathogen species isolated from the giant panda to detect antimicrobial activity, i.e., *E*. *coli*, *Staphylococcus aureus*, and *Salmonella enterica*. We also used reference strains, including *E*. *coli* (CCTCC AB 212358), *Staphylococcus aureus* (CCTCC AB 91053), and *Salmonella enterica* (CCTCC M 95026), purchased from the China Center for Type Culture Collection (CCTCC). The antimicrobial abilities of surfactin were assessed using the Oxford Cup method [14]. Briefly, 50 μL of each pathogen was coated on LB solid medium and diluted to 1 × 10^7^ CFU/mL with the Oxford Cup. Ten microliters of the HPLC-purified extract was diluted and added at the midpoint of the Oxford Cup. The samples were then cultivated at 37°C for 24 h, and the inhibition zone was measured. Methanol was used as a control. Inhibition zones for each group were measured three times to reduce experimental error.

For analysis of resistance to temperature, pH, and proteases, 0.4 mg/mL surfactin extract samples were processed at 60–121°C in a water bath for 30 min, at pH 1–12 overnight (with the pH adjusted using HCl or NaOH), or with 1–3 mg/mL trypsin (pH = 8) or pepsin (pH = 2; Sangon Biotech Co., Ltd., Shanghai, China) for 4 h. After treatment, the processed surfactin extract was adjusted to the control environment (37°C, pH = 7), and antimicrobial activity was then assessed as previously described.

### Statistical analysis

The expression and antimicrobial effects of surfactin in each group were evaluated three times to reduce error. Significant differences were determined by analysis of *P* values using t-tests in IBM SPSS statistics software (version 20.0).

## Results

### Identification of strains and surfactin from *B*. *subtilis* HH2

The strain *B*. *subtilis* HH2 used in the current study were first identified based on 16S rRNA and gyrA genes. The sequencing results are shown in S1 Table. It is hard to determine the strain at subspecies level, since there were several *B*. *subtilis* subspecies showed more than 99% homology with our strain, e.g. *B*. *subtilis* subsp. subtilis strain SRCM100333, *B*. *subtilis* subsp. spizizenii ATCC 6633, according to gyrA gene BLAST results.

**Fig 1:**
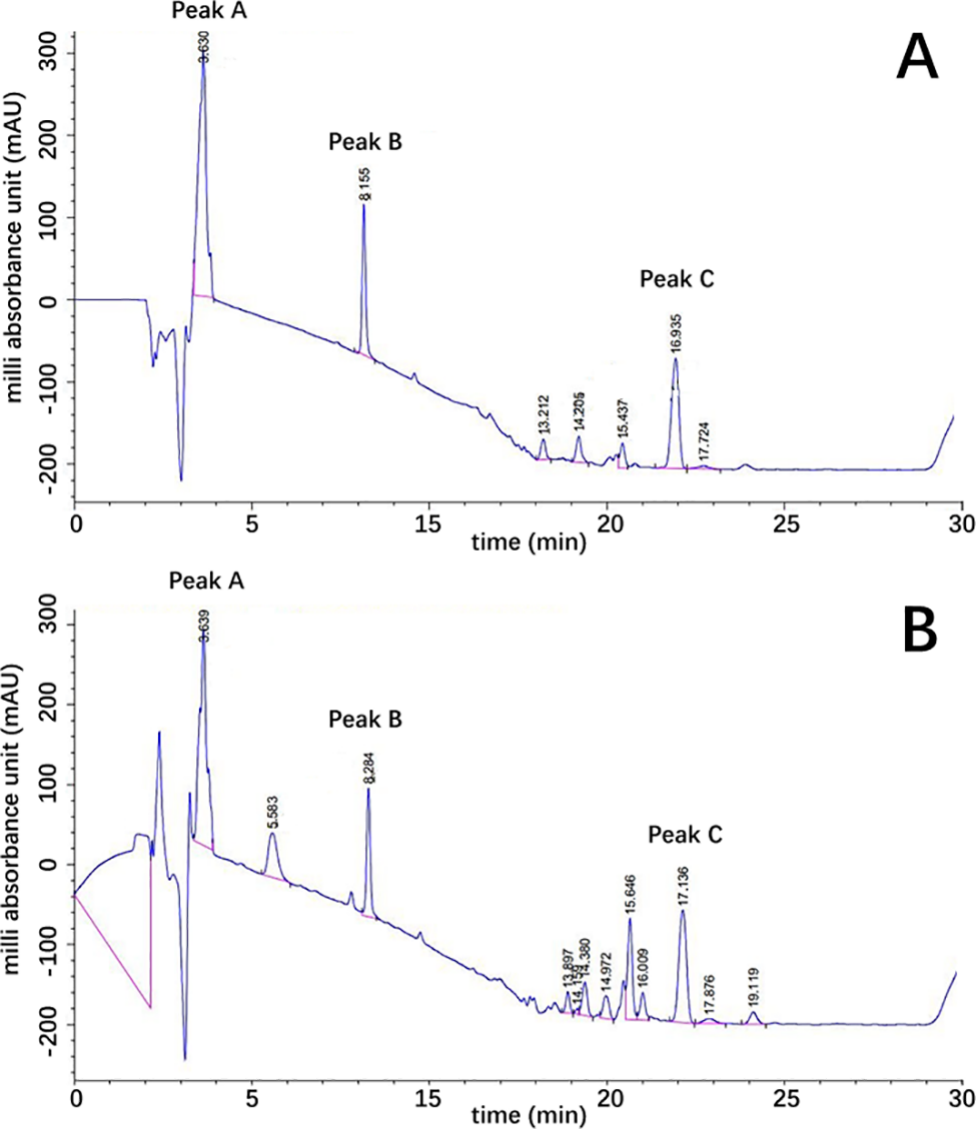
High-performance liquid chromatography (HPLC) results of (A) commercial surfactin sample, and (B) our extract surfactin of *B*. *subtilis* HH2 in LB medium. There were three main peaks (Peak A-C) of the extract and the surfactin standard in the same location.

The surfactin gene *sfp* was also identified by PCR and RT-PCR from the *B*. *subtilis* HH2 genome and transcriptome, respectively. Sequencing of the PCR product by BLAST search in the NCBI database showed that the gene had 99% homology with *sfp* (X63158.1), indicating that this bacterium had the ability to produce surfactin. Next, we used HPLC technology to evaluate surfactin extracts from metabolites of *B*. *subtilis* and compared the results with those of commercial surfactin. From this analysis, three peaks of the extract had the same location as the surfactin standard; however, several additional peaks were also observed (Fig 1). The additional peaks were discarded for purification of the extract for subsequent analysis since they may represent another substance.

### Expression of surfactin from *B*. *subtilis* HH2 in the presence of different concentrations of cellulose

**Fig 2:**
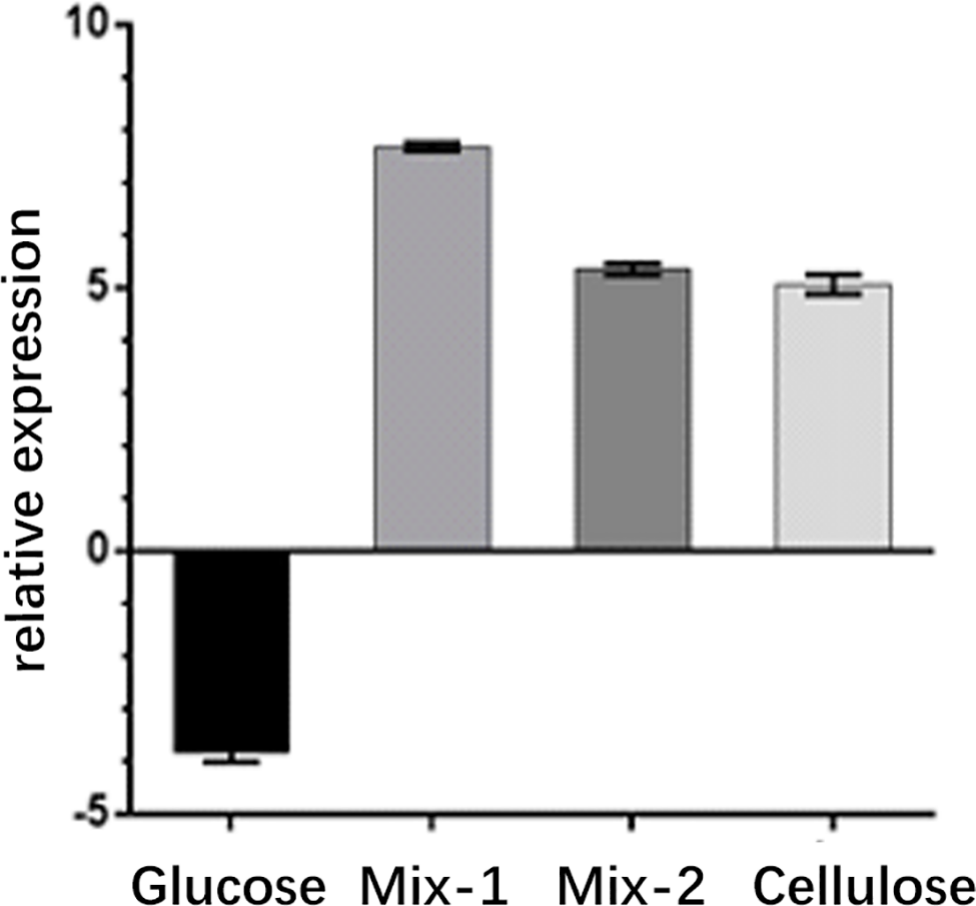
The relevant expression of surfactin of *B*. *subtilis* HH2 in four different media by RT-qPCR.

To further explore surfactin production by *B*. *subtilis* in the presence of high concentrations of cellulose, which is normally observed in the herbivore intestine, we used qPCR and HPLC to compare the expression levels of surfactin in the presence of different cellulose concentrations. Using RT-qPCR, we found that cells grown in the presence of 1% glucose expressed the least *sfp*, whereas cells in the mixed-1 group exhibited the highest expression, followed by cells in mixed-2 group and then the cellulose group (Fig 2).

The results of HPLC were similar to those of qPCR, with the areas of the three peaks showing the following pattern: mixed-1 group > mixed-2 group > cellulose group > glucose group (Table 1). These data further confirmed that an appropriate concentration of cellulose increased surfactin expression.

**Table 1:** Production of surfactin (mg/L).

Groups	Peak A	Peak B	Peak C	Gross
Glucose	94	19.8	28.2	142
Mix-1	126.6	42	103.4	272
Mix-2	98.9	29.9	54.4	183.2
Cellulose	117.7	15.8	36.6	170.1

### Characterization of the antimicrobial activity and resistance of surfactin

**Fig 3:**
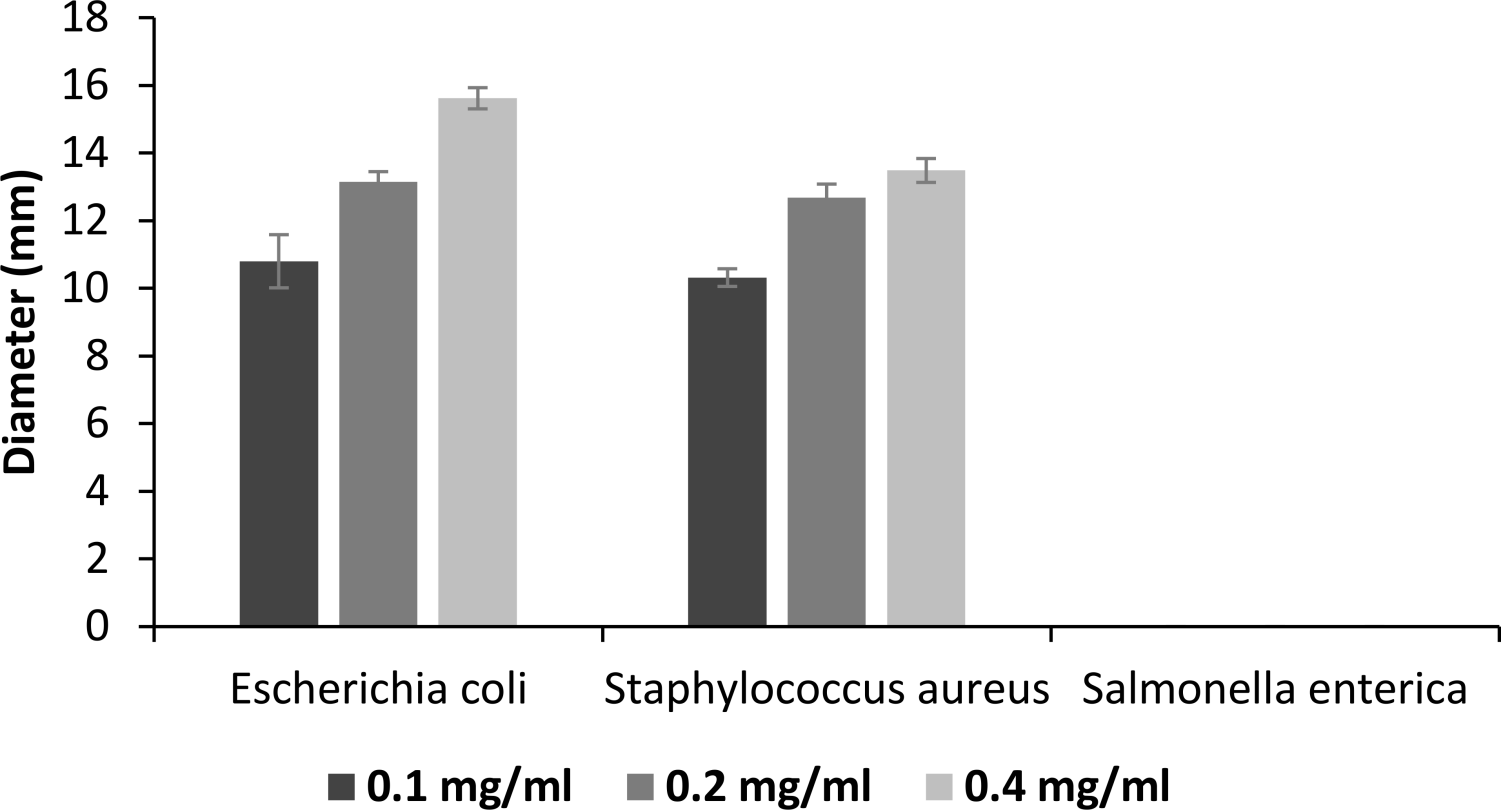
The antimicrobial ability of the surfactin in the concentrate of 0.1–0.4 mg/mL against three intestinal common pathogens (*Escherichia coli*, *Staphylococcus aureus*, and *Salmonella enterica*), which indicated by the inhibition zone assessed by Oxford Cup method.

After identification of surfactin expression in *B*. *subtilis*, the antimicrobial activity of surfactin extracts and its resistance to temperature, pH, and proteases were analyzed. Because most pathogens may not show optimal growth condition in a cellulose environment, we used standard culture conditions for analysis of the antimicrobial activity of the protein. From the results, we found that surfactin secreted by *B*. *subtilis* HH2 showed high inhibitory activity against all the strains of *E*. *coli* and *Staphylococcus aureus*. This inhibitory ability increased as the concentration of surfactin increased, reaching a peak at 0.4 mg/mL surfactin. However, surfactin was not sufficiently effective for inhibition of *Salmonella enterica*. For *Salmonella enterica* strains, no inhibition zone was observed in any group (Fig 3). Because *E*. *coli* was the most sensitive to surfactin, we used the reference strain *E*. *coli* CCTCC AB 212358 and 0.4 mg/mL surfactin for subsequent pressure-resistance tests.

**Fig 4:**
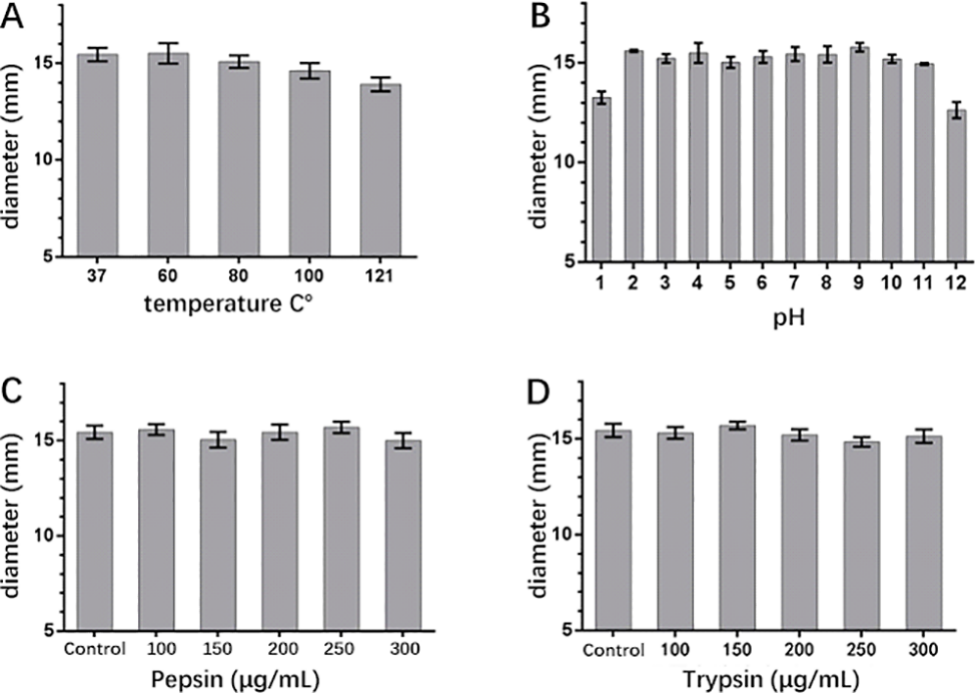
The antimicrobial-ability resistance of surfactin under a rouge of harsh elements including (A) temperature (37–121°C), (B) pH (1–12), (C) pepsin (100–300 μg/mL), and (D) trypsin (100–300 μg/mL). The antimicrobial inhibition zone was measured under 0.4 mg/mL surfactin against to the indictor strain (*E*. *c*oli CCTCC AB 212358).

Notably, the surfactin extract from *B*. *subtilis* HH2 showed a good stability from 60 to 100°C, with no significant difference in antibacterial activity for *E*. *coli* in the control group at 37°C (*P* > 0.05). However, the antibacterial ability significantly decreased when the temperature reached 121°C (*P* < 0.05). Additionally, the surfactin extract maintained most of its antibacterial activity within the pH range of 2–11 compared with that at pH 7 (*P* > 0.05); however, there was a significant decrease in activity at pH 1 and 12 (*P* < 0.05). In terms of protease resistance, the antimicrobial activity of surfactin extract did not show any significant changes compared with the control group (no protease; *P* > 0.05), even at the highest concentration of trypsin or pepsin (300 μg/mL; Fig 4).

## Discussion

Several lipopeptides secreted by *B*. *subtilis* can confer strong antipathogenic effects and thus benefit the host by balancing the intestinal microbiome [15, 16]. Among them, iturins, fengycin, and surfactin were the most thorough of the research [17]. Iturins and fengycin could suppress many fungi microsclerotial germination and may induce plant defense responses as activators, but have their limit in a specific anti-bacterial spectrum [18–21]. However, surfactin shows antimicrobial activities in the nanomolar range against a broad spectrum of bacteria and fungi, in the meantime, has anti-inflammatory activity such as lipopolysaccharide-activated macrophages, which is more effective for animal [20, 22].

In the panda intestine, the concentration of cellulose is high because the main food consumed by this animal, i.e., bamboo, contains approximately 41.8% cellulose [23], resulting in a harsh gut environment and marked inhibition of bacterial growth [24]. Therefore, the efficient secretion ability of antibacterial peptides in a cellulose environment should be considered when developing herbivore probiotics. Additionally, host species specificity is important when selecting probiotics because probiotics from heterologous animals may show different adhesion and reproductive capacities in the treated host intestine, which may increase the risk of negative physiological effects [6, 25, 26]. Here, we used *B*. *subtilis* HH2 original from the giant panda as a model to determine the expression and antimicrobial characteristics of surfactin in the presence of different concentrations of cellulose. Our results showed that an appropriate cellulose environment may increase the expression of surfactin secreted by *B*. *subtilis* HH2. These findings provided insights into the potential functions of herbivore intestinal probiotics.

Generally, glucose culture condition can be beneficial for the expression of most genes and can promote bacterial growth and division. In a previous study, we showed that cellulose was not an ideal carbon source for *B*. *subtilis* HH2; most nonessential genes (e.g., genes involved in chemotaxis and motility) in the cellulose group were down-regulated compared with that in the glucose group in order to conserve energy [7]. However, in this study, we found that *B*. *subtilis* HH2 showed optimal growth in a glucose medium, accompanied by low surfactin expression. Conversely, the appropriate concentration of cellulose (0.33% cellulose and 0.67% glucose) could increase surfactin expression. Therefore, the growth pressure of cellulose may induce the expression of surfactin. Previous studies have shown that bacterial growth in a stressful environment, e.g., in the presence of fibers, fever, and high salt, can result in selective upregulation of some genes to resist the harsh environmental conditions. The protective effects include the secretion of certain proteins to protect cells [27], secretion of antibiotics and other chemicals to inhibit competing microbial survival [28], and secretion of hydrolytic enzymes to remove extracellular proteins and polysaccharides [29]. In an appropriate cellulose environment, *B*. *subtilis* HH2 may increase surfactin expression to inhibit other bacteria and pathogens; thus, this bacterium could yield more nutritional substances and benefit host intestinal health.

In our study, surfactin produced by *B*. *subtilis* HH2 showed effective inhibitory activity against *E*. *coli* and *Staphylococcus aureus*, with concentration-dependent effects. One of the most important effect mechanism of the lipopeptide surfactin is leakage and lysis of lipid membranes, which may explain Gram-negative bacteria *E*. *coli* was more sensitive than Gram-positive bacteria *Staphylococcus aureus* [30]. In general, the effects of surfactin on these two bacterial species may provide insights into its applications in biocontrol of herbivore mammal intestinal pathogens as an alternative to antibiotics. However, we did not detect significant inhibitory activity against *Salmonella enterica*. Compared with the commercial standard, our crude extract of surfactin showed some extra peaks and impurities when analyzed by HPLC. Therefore, for more precise calculations, we discarded all additional components and may have also removed some antimicrobial substances and components, thus yielding a different antibacterial spectrum for HH2 compared with standard strains [31]. On the other hand, previous studies indicated that surfactin secreted by different *B*. *subtilis* strains may have divergent antimicrobial abilities. For example, Mireles et al. [32] indicated that surfactin from *B*. *subtilis* has good antibacterial effects against *Salmonella enterica*, *E*. *coli*, and *Proteus mirabilis*; however, Loiseau et al. [12] showed that surfactin can only inhibit *Legionella* strains but does not affect any other strains, including *Salmonella enterica*.

Another advantage of surfactin produced by microorganisms is that the protein shows biodegradability under conditions of extreme temperature and pH [33]. In our study, surfactin from *B*. *subtilis* HH2 maintained its antimicrobial activity after being exposed to a range of stresses, including high temperature, extreme pH, and two different proteases. Notably, the surfactin from *B*. *subtilis* HH2 showed good stability at high temperatures, even as high as 80–100°C, indicating that this protein may have potential applications for industrial production because food additives may require high temperatures during manufacturing. The stability of surfactin can be attributed to its saddle-shape, which gives it a close-knit structure [34]; this is essential for maintaining host intestinal health and allows the protein to remain active under various stress conditions in the gastrointestinal tract of animals.

In summary, we demonstrated that a panda-derived *B*. *subtilis* HH2 strain enhanced surfactin secretion under conditions of suitable cellulose concentrations, resulting in efficient antimicrobial activity in a variety of environmental conditions. These findings expand our knowledge of surfactin production by herbivore probiotic strains in the presence of cellulose as a carbon source and provide insights into the development of potential panda-specific probiotics. Further studies are required to determine the specific mechanisms mediating the expression of surfactin as a probiotic in an appropriate cellulose environment.

## Supporting information

S1 Table The gene sequencing results. (XLSX)
